# Management of anaphylaxis in schools: Evaluation of an epinephrine auto-injector (EpiPen®) use by school personnel and comparison of two approaches of soliciting participation

**DOI:** 10.1186/1710-1492-8-4

**Published:** 2012-07-09

**Authors:** Nha Uyen Nguyen Luu, Lisa Cicutto, Lianne Soller, Lawrence Joseph, Susan Waserman, Yvan St-Pierre, Ann Clarke

**Affiliations:** 1 Department of Epidemiology and Biostatistics, McGill University, 687 Pine Avenue West, V Building, Room V1.09, Montreal, Quebec, H3A 1A1, Canada; 2 Department of Medicine, Division of Clinical Immunology and Allergy, University of Montreal, Montreal, Quebec, Canada; 3 Department of Medicine, National Jewish Health, Denver, CO, USA; 4 Department of Medicine, Division of Clinical Epidemiology, McGill University Health Center, Montreal, Quebec, Canada; 5 Department of Medicine, Division of Clinical Immunology and Allergy, McMaster University, Hamilton, ON, Canada; 6 Department of Medicine, Division of Clinical Immunology and Allergy, McGill University Health Center, Montreal, Quebec, Canada

**Keywords:** Anaphylaxis, Epinephrine, Food allergy, School, Treatment, Selection bias, Consent bias, Volunteer bias

## Abstract

**Background:**

There has been no large study characterizing selection bias in allergy and evaluating school personnel’s ability to use an epinephrine auto-injector (EpiPen®). Our objective was to determine if the consent process introduces selection bias by comparing 2 methods of soliciting participation of school personnel in a study evaluating their ability to demonstrate the EpiPen®.

**Methods:**

School personnel from randomly selected schools in Quebec were approached using a 1) *partial* or 2) *full disclosure* approach and were assessed on their ability to use the EpiPen® and identify anaphylaxis.

**Results:**

343 school personnel participated. In the *full disclosure* group, the participation rate was lower: 21.9% (95%CI, 19.0%-25.2%) versus 40.7% (95%CI, 36.1%-45.3%), but more participants achieved a perfect score: 26.3% (95%CI, 19.6%-33.9%) versus 15.8% (95%CI, 10.8%-21.8%), and identified 3 signs of anaphylaxis: 71.8% (95%CI, 64.0%-78.7%) versus 55.6% (95%CI, 48.2%-62.9%).

**Conclusions:**

Selection bias is suspected as school personnel who were fully informed of the purpose of the assessment were less likely to participate; those who participated among the fully informed were more likely to earn perfect scores and identify anaphylaxis. As the process of consent can influence participation and bias outcomes, researchers and Ethics Boards need to consider conditions under which studies can proceed without full consent. Despite training, school personnel perform poorly when asked to demonstrate the EpiPen®.

## Background

Food allergy is a serious condition affecting 3.9% of children in the United States [1], and can lead to systemic life-threatening symptoms or anaphylaxis [2]. There is currently no well-established curative treatment for food allergy and management relies on avoidance and therapy with epinephrine for reactions caused by accidental exposures [3]. We and others have shown that despite increasing societal awareness of the potentially fatal consequences of food allergy, accidental exposures continue to occur [4-8] and about 10% of fatal food-associated anaphylactic reactions take place in school [9,10]. As school represents a situation where parents must rely on other caregivers to respond to a severe allergic reaction, school personnel must be able to recognize anaphylaxis and know how to administer epinephrine using an auto-injector device such as the EpiPen® [11]. A delay in epinephrine administration substantially increases the risk for fatality [10,12,13].

Previous research has shown that school personnel are not well prepared to recognize and treat food-induced allergic reactions and anaphylaxis [14,15]. However, there has not been any large study evaluating the ability of school personnel to administer an epinephrine auto-injector such as the EpiPen®. A brief report in 2005 showed that only 12% of 100 elementary school teachers in Ontario, Canada correctly demonstrated the use of the EpiPen® [16]. However, in a recent electronic survey of 724 school teachers in Canada (published only in abstract form), 82% of the respondents reported receiving training from health professionals or parents in using the auto-injector and 80% claimed they were confident in using it [17]. In contrast to the Ontario experience, these teachers were not actually evaluated on their ability to use the auto-injector. As part of a Canadian-wide study examining the influence of different provincial policies on the recognition and management of anaphylaxis in schools [18], our research team assessed the ability of school personnel in Quebec to demonstrate the EpiPen® technique and identify symptoms of anaphylaxis. In the other Canadian provinces, school personnel were fully informed of the purpose of the assessment (i.e., *full disclosure*). However, it was anticipated that such *full disclosure* may result in volunteer or consent bias, a form of selection bias where those who volunteer or consent to participate differ from those who do not, leading to an incorrect assessment of performance capacity [19-21]. In Quebec, we explored the potential role of volunteer or consent bias by approaching school personnel in 2 different ways: 1. A *partial disclosure* approach in which school personnel were not notified in advance of the EpiPen® demonstration and 2. A *full disclosure* approach in which school personnel were informed in advance that they would have to demonstrate the use of the EpiPen®. In this manuscript, the participation rates and outcomes of these 2 groups were compared to determine if a volunteer or a consent bias was present.

## Methods

### Selection of participants

In 2008, 2 school boards out of 10 within 1 h of traveling time from downtown Montreal, Quebec were randomly selected. Initially, 20 schools, including elementary (kindergarten – grade 6) and secondary schools (grade 7 – 11), were randomly identified within each selected school board in a 4:1 ratio, representing the ratio of elementary to secondary schools in Quebec [22]. Following the approval by school boards and the principals of the selected schools, the school secretary was contacted to arrange a time for the assessors to visit the school. Once a time was arranged, the research team provided invitations (detailing date, time, location) to the school secretary for distribution to all school staff. If a school board or a school refused to participate or did not provide an answer within 6 months after multiple contacts, another one was randomly selected to replace it. All school personnel, including teachers, lunch monitors, administrative staff, school nurses, and janitors, were invited to participate.

### Methods of approaching school personnel

In the other Canadian provinces where the ability of school personnel to recognize anaphylaxis and administer an epinephrine auto-injector was also assessed, the investigators were required by their Research Ethics Boards to fully disclose the purpose of the assessor’s visit in advance to participating school personnel. However, because we suspected that such an approach might introduce bias, in Quebec, the *partial* and *full disclosure* approaches were compared. One of the selected school boards was randomly assigned to a *partial disclosure* approach, i.e. school personnel and school contacts were not informed in advance of the EpiPen® demonstration and were told in the study invitation that the investigators were studying school personnel’s knowledge “regarding allergies and how schools are prepared for children with allergies.” The 2nd school board was assigned to a *full disclosure* approach: school personnel were informed in the study invitation of the exact purpose of the assessor’s visit, i.e., they would be asked to “show how they use an EpiPen® to help students with life-threatening allergies (anaphylaxis).” Both groups were told in the invitation that they would be provided “feedback, education, and materials on helping students with allergies in school.” For both groups, on the day of the visit, prior to the assessment, all school personnel who were interested presented themselves to the assessor and were requested to sign an informed consent which informed them that they would be asked to demonstrate the use of the EpiPen®. It should be noted that no participants in the *partial disclosure* group refused to participate at this stage.

### EpiPen® assessment

Although there are 2 epinephrine auto-administration devices on the market in Canada, the Twinject® was only introduced in the fall of 2005 and has had relatively limited uptake. In addition, the Quebec Ministry of Health and Social Services stipulated that school personnel that are not trained health care workers are not allowed to administer the 2^nd^ dose of the Twinject® [23], making this device less favored in the school environment. Therefore, it was decided to only assess the EpiPen® technique. Since we have completed the assessment of the school personnel, the company producing the EpiPen® has released a new EpiPen® device, which has a slightly different shape and color. However, the steps required to use the EpiPen® remain the same.

The assessors visited schools between October 2008 and May 2009. The assessment visit for each school was concluded within one day; there were no repeat visit to schools. A location in the school was secured to allow for privacy and a one-to-one interface with the assessor. Assessors were trained nurses or allergists, and their technique was assessed and ensured for accuracy.

EpiPen® technique was assessed based on accurate completion of 4 steps [24,25] using an auto-injector demonstrator:

1. Removal of the grey safety cap;

2. Placement of the black tip against the mid-outer thigh;

3. Application of firm pressure until the device activates (“click” heard);

4. Holding of the device in place for 10 s.

To calculate a score for each participant, one point was assigned for successful completion of each step (maximum 4 points).

Participants were also asked to verbally provide 3 symptoms or signs of anaphylaxis. The 1^st^ 3 symptoms or signs mentioned by the participant were recorded by the assessor. The answers were evaluated using previously published work on the definition and symptoms and signs of anaphylaxis [26]. After the assessor evaluated the participant’s EpiPen® technique and the participant answered questions regarding previous training and indications for administration of an epinephrine auto-injector, the assessor provided feedback on the participant’s technique and coaching until accurate technique was achieved.

### Statistical analysis

Descriptive statistics were compiled for all variables. The participation rate was defined as the number of school personnel who participated divided by the estimated number of school personnel as provided by school secretaries. Data were analyzed according to each step of the EpiPen® technique regarding whether or not the step was performed accurately and an overall accuracy score was calculated. School personnel with a *partial disclosure* approach were compared to those with a *full disclosure* approach in terms of participation rate, scores and capacity to provide indications for epinephrine administration and the difference between groups and confidence intervals (CI) were reported. Multivariate logistic regression analyses were used to determine if the method of approach (i.e., *partial* versus *full* disclosure) was associated with accurate demonstration of the EpiPen® technique after adjustment for confounders. Potential confounders considered included type of school (elementary or secondary), prior training of the school personnel in the use of the EpiPen®, prior training by a nurse, and prior training using an EpiPen® trainer. These analyses were adjusted for clustering of participants within schools by allowing the variance to differ between schools.

This study was approved by the Research Ethics Board of the McGill University Health Center.

## Results

In the *partial disclosure* group*,* 33 schools were approached and 9 schools participated (7 elementary, 2 secondary); 460 personnel were approached and 187 participated (40.7%, 95% CI, 36.1%-45.3%). In the *full disclosure* group*,* 34 schools were approached and 11 participated (9 elementary, 2 secondary); 711 personnel were approached and 156 participated (21.9%, 95% CI, 19.0%-25.2%) (Table 1).

**Table 1:** Participation rates

	**Partial and Full Disclosure Groups**	**Partial Disclosure Group**	**Full Disclosure Group**	**Difference** **% (95% CI)**
**Overall**				
** Participants**	343	187	156	
** School personnel** **approached**	1171	460	711	
** Participation rate %**	29.3	40.7	21.9	18.7 (13.3, 24.1)
**Elementary schools**				
** Participants**	258	130	128	
** School personnel** **approached**	805	334	471	
** Participation rate %**	32.0	38.9	27.2	11.7 (5.2, 18.3)
**Secondary schools**				
** Participants**	85	57	28	
** School personnel** **approached**	366	126	240	
** Participation rate %**	23.2	45.2	11.7	33.6 (24.0, 43.2)

CI – Confidence Interval.

The majority of participants in both groups were teachers: 64.2% in the *partial disclosure* group and 66.7% in the *full disclosure* group (Table 2). Among all participants, 18.1% were lunch monitors and 11.7% were administrative staff; only 3 school nurses (0.9%) participated. The vast majority of participants in both groups (89.2%) reported previous training, most of them having been trained by school nurses (93.8%). The training involved practice with an EpiPen® demonstrator for 76.1% in the *partial disclosure* and 54.9% in the *full disclosure* group. More school personnel from elementary schools reported training: 91.5% versus 82.4%, including training with an EpiPen® demonstrator: 75.7% versus 34.3%.

**Table 2:** Characteristics of school personnel and training

**Characteristics**	**All participants** **N = 343** **%**	**Partial Disclosure Group** **N = 187** **%**	**Full Disclosure Group** **N = 156** **%**
**School personnel**^**1**^			
** Teachers**	65.3	64.2	66.7
** Lunch monitors**	18.1	21.9	13.5
** Administrative staff**	11.7	10.2	13.5
** Nurses**	0.9	1.6	0.0
** Others**	2.0	1.6	2.6
**Personnel from elementary schools**	75.2	69.5	82.1
**Prior training**			
** Overall**	89.2	87.2	91.7
** Elementary schools**	91.5	91.5	91.4
** Secondary schools**	82.4	77.2	92.9
**Prior training by nurse**			
** Overall**	93.8	95.1	92.3
** Elementary schools**	94.5	95.0	94.0
**Prior training using an EpiPen® demonstrator**			
** Secondary schools**	91.4	95.5	84.6
** Overall**	66.2	76.1	54.9
** Elementary schools**	75.7	88.2	62.9
** Secondary schools**	34.3	43.2	19.2

N – Number of participants.

^1^ These data are missing for one school.

The mean scores for the EpiPen® assessment were 2.52 (95% CI, 2.39-2.65) in the *partial disclosure* group versus 2.64 (95% CI, 2.46-2.83) in the *full disclosure* group (Table 3). Overall, only 20.6% of participants had a perfect 4 point score. Participants from the *full disclosure* group were more likely to have a perfect score: 26.3% (95% CI, 19.6%-33.9%) versus 15.8% (95% CI, 10.8%-21.8%). Mean scores were also higher in elementary schools: 2.67 (95% CI, 2.55-2.80) versus 2.28 (95% CI, 2.04-2.51) in secondary schools, and school personnel from elementary schools were more likely to earn a perfect score: 23.7% (95% CI, 18.7%-29.4%) versus 10.8% (95% CI, 5.1%-19.6%) in secondary schools (Table 4).

**Table 3:** School personnel scores for EpiPen® assessment (Partial versus Full disclosure Groups)

**Score**	**All participants** **N = 343** **%**	**Partial Disclosure Group** **N = 187** **% (95% CI)**	**Full Disclosure Group** **N = 156** **% (95% CI)**	**Difference** **% (95% CI)**
**0**	3.5	1.6 (0.3, 4.7)	5.8 (2.7, 10.7)	−4.1 (−8.2, 0.0)
**1**	10.0	7.6 (4.2, 12.4)	12.8 (8.0, 19.1)	−5.2 (−11.7, 1.3)
**2**	32.4	43.5 (36.2, 51.0)	19.2 (13.4, 26.3)	24.2 (14.8, 33.7)
**3**	33.5	31.5 (24.9, 38.8)	35.9 (28.4, 44.0)	−4.4 (−14.5, 5.7)
**4**	20.6	15.8 (10.8, 21.8)	26.3 (19.6, 33.9)	−10.5 (−19.2, -1.8)
**Mean Score** **(95% CI)**	2.58	2.52 (2.39, 2.65)	2.64 (2.46, 2.83)	−0.12 (−0.34, 0.11)

N – Number of participants.

CI – Confidence Interval.

**Table 4:** School personnel scores for EpiPen® assessment (Elementary versus Secondary schools)

**Score**	**All participants** **N = 343** **%**	**Elementary schools** **N = 258** **% (95% CI)**	**Secondary schools** **N = 85** **% (95% CI)**	**Difference** **% (95% CI)**
**0**	3.5	1.9 (0.6, 4.5)	8.4 (3.5, 16.6)	−6.5 (−12.7, -0.3)
**1**	10.0	9.3 (6.1, 13.6)	12.0 (5.9, 21.0)	−2.7 (−10.6, 5.1)
**2**	32.4	31.9 (26.3, 38.0)	33.7 (23.7, 44.9)	−1.8 (−13.5, 9.8)
**3**	33.5	33.1 (27.4, 39.2)	34.9 (24.8, 46.2)	−1.9 (−13.6, 9.9)
**4**	20.6	23.7 (18.7, 29.4)	10.8 (5.1, 19.6)	12.9 (4.4, 21.4)
**Mean Score** **(95% CI)**	2.58	2.67 (2.55, 2.80)	2.28 (2.04, 2.51)	0.4 (0.13, 0.66)

N – Number of participants.

CI – Confidence Interval.

The multivariate logistic regression analysis showed that a *full disclosure* approach remained associated with a perfect score after adjustment for confounders: Odds Ratio (OR) 2.6 (95% CI, 1.5-4.6). Prior training with an EpiPen® demonstrator was also associated with accurate demonstration of the EpiPen® technique: OR 5.3 (95% CI, 2.6-10.7).

When considering the percentage of participants correctly demonstrating each step of the EpiPen® technique (Table 5), there was no between group difference for steps 1 (removal of the safety cap) and 4 (holding the device in place for 10 s). However, those in the *full disclosure* group were slightly more likely to perform step 2 (placement of the black tip against the mid-outer thigh) correctly: 59.6% (95% CI, 51.5%-67.4%) versus 45.1% (95% CI, 37.8%-52.6%). In contrast, those in the *partial disclosure* group were slightly more likely to perform step 3 (application of firm pressure until the device activates) correctly: 91.3% (95% CI, 86.3%-94.9%) versus 82.1% (95% CI, 75.1%-87.7%). However, because it is not known if keeping the EpiPen® device against the thigh for 10 s (step 4) is really necessary to ensure efficacy, we also calculated participants’ scores based on accurate completion of the first 3 steps described above. When this last step is omitted, those in the *full disclosure* group were more likely to complete steps 1 through 3 correctly: 51.3% (95% CI, 43.3%-59.4%) versus 31.0% (95% CI, 24.4%-38.2%).

**Table 5:** School personnel’s ability to complete each step of the EpiPen® assessment

	**All participants** **N = 343** **%**	**Partial Disclosure Group** **N = 187** **% (95% CI)**	**Full Disclosure Group** **N = 156** **% (95% CI)**	**Difference** **% (95% CI)**
**Step 1**	81,5	81.0 (74.6, 86.4)	82.1 (75.1, 87.7)	−1.1 (−9.3, 7.2)
**Step 2**	51,8	45.1 (37.8, 52.6)	59.6 (51.5, 67.4)	−14.5 (−25.0, -4.0)
**Step 3**	87,1	91.3 (86.3, 94.9)	82.1 (75.1, 87.7)	9.3 (2.0, 16.5)
**Step 4**	37,2	34.6 (27.8, 41.9)	40.4 (32.6, 48.5)	−5.8 (−16.1, 4.5)

CI – Confidence Interval.

Step 1 - Removal of the grey safety cap.

Step 2 - Placement of the black tip against mid-outer thigh.

Step 3 - Application of firm pressure until the devices activates (“click” heard).

Step 4 - Holding of the device in place for 10 s.

Overall, 63% of participants were able to identify 3 signs or symptoms of anaphylaxis that should prompt the administration of epinephrine, more in schools with *full disclosure:* 71.8% (95% CI, 64.0%-78.7%) versus 55.6% (95% CI, 48.2%-62.9%).

## Discussion

In this study, we explored the existence of volunteer or consent bias by using 2 different methods to solicit the participation of school personnel in research evaluating competency in the use of an epinephrine auto-injector (EpiPen®): *partial disclosure* and *full disclosure*. The participation rate was higher in the *partial disclosure* group (between group difference 18.7%, 95% CI, 13.3%-24.1%) and participants from the *full disclosure* group were more likely to earn a perfect score (between group difference 10.5%, 95% CI, 1.8%-19.2%), demonstrate the 3 critical steps correctly (between group difference 20.3%, 95% CI, 10.0%-30.6%), and identify signs of anaphylaxis (between group difference 16.2%, 95% CI, 6.2%-26.2%). These results suggest the existence of a volunteer or consent bias, a form of selection bias where individuals who volunteer for a study may have specific characteristics that distinguish them from non-volunteers and that may affect outcomes; for example, participants may be more likely to find the topic interesting and usually expect to be evaluated positively [27]. In our study, school personnel from the *partial disclosure* group were not given all the information about the purpose of the study and the EpiPen® assessment prior to the assessors’ visit. Consequently, they were unlikely to be reluctant to participate because of concerns regarding their knowledge and competence, but their performance was generally poorer. In contrast, those in the *full disclosure* group were completely aware of the purpose of the assessment and those with a greater interest and possibly knowledge in the topic were more willing to participate, leading to an overestimation of competence relative to the general population. It is also possible that those who chose to participate also practised or prepared prior to the evaluation, enhancing their performance. This suggests that the timing and the process of informed consent can affect the participation rate and the interpretation of the results. Although this threat to the validity of a study that arises from the consent process has been described previously [20,21,28,29], we are the first to explore its influence in allergy research.

In comparing the 2 approaches, we tried to ensure that the school boards were as similar as possible other than in the detailing of the consent by randomly selecting school boards of similar size in the same urban area. In addition, in Quebec, as school nurses responsible for school personnel training are employed by the Ministry of Health and Social Services and not by individual school boards, the EpiPen® training is less likely to be influenced by school board environments and likely to be reasonably similar throughout the province. Further, we adjusted for possible differences between the *partial* and *full disclosure* groups through regression analyses and demonstrated that the *full disclosure* group continued to perform more favourably. However, it is possible that the school boards differed in ways we did not consider or were unable to measure and these differences influenced the performance of school personnel. It should be noted that in the multivariate analysis, prior training with an auto-injector and being in the *full disclosure* group were independent predictors of a perfect score. Hence, although fewer in the *full disclosure* group reported training than those in the *partial disclosure* group, they still performed better and we anticipate that had more in the *full disclosure* group reported training, the between group difference in performance would be even greater.

It is also possible that there was contamination within and between groups. As it was not feasible to conduct all school assessments on the same day, assessments were staggered over an 8-month period. Hence, it is possible that school personnel within the *partial* or *full disclosure* group assessed early in the process communicated with those in the *partial disclosure* group who were assessed later, informing them of the purpose of the assessment. Such contamination would likely minimize our between group difference and make our assessment of selection bias conservative. In addition, our analyses were adjusted to take into account the grouping of participants by school, and we found that the effects of within-school versus between-school variations were not significant, as the size of the confidence intervals was only minimally affected. Although it was not the purpose of this small study, it would have been interesting to compare participants and non-participants in terms of their anaphylaxis interest and knowledge to better characterize the bias illustrated in this study.

Our results reporting that only 26.3% (95% CI, 19.6%-33.9%) among the *full disclosure* group are able to accurately demonstrate the use of the EpiPen® are disturbing as they likely overestimate the competence of school personnel. The 15.8% (95% CI, 10.8%-21.8%) demonstrating correct usage in the *partial disclosure* group is likely more representative, but it, too, is probably an overestimate as the most informed were still more likely to participate even in this group. Although personnel in elementary schools performed more favourably, possibly because they feel younger children are more reliant on them, only 23.7% (95% CI, 18.7%-29.4%) were able to correctly use the EpiPen®. These results are worrisome because it has been shown that inability to use an epinephrine auto-injector may contribute to a delay in the treatment of anaphylaxis [11,30] which can increase the risk for fatality [10,12].

Given the poor performance observed despite 89.2% of all participants reporting training, the quality and frequency of school personnel training needs to be examined. In Quebec, school personnel are trained in allergy and anaphylaxis management and EpiPen® use on a regular basis [31]. However, the content and frequency of training programs may vary as there are no provincial guidelines. In our study, training involving an EpiPen® demonstrator was associated with better performance. Other authors have also recommended use of the auto-injector training device and frequent review to increase knowledge retention [11,15]. A training model using an audio-visual presentation and written material on anaphylaxis and epinephrine administration followed by a meeting with allergic children was developed for school personnel in San Francisco in 2004, and significantly increased knowledge and perceived self-efficacy in 53 participants [32]. Such a training model could be adapted and studied in Canada.

## Conclusions

Although Research Ethics Boards usually ask investigators to fully disclose the intended purpose of their research to potential participants, we have shown that the process of consent can influence participation and bias outcomes. Investigators need to appreciate and acknowledge the potential bias that may be introduced by the consent process and attempt to fulfill ethical requirements while minimizing bias. While respecting participants’ rights, ethical issues regarding the consent process have to be discussed with Research Ethics Boards whenever the scientific validity of results may be compromised. Researchers and Ethics Boards may need to be educated on circumstances under which studies can proceed without full prior disclosure. Further, we have shown that despite being trained to recognize anaphylaxis and to administer epinephrine, school personnel perform poorly when asked to demonstrate how to use the EpiPen®. The content, quality and frequency of allergy and anaphylaxis training programs for school personnel have to be re-examined. As recommended by numerous guidelines [33-35] and required by legislation in at least one Canadian province [36], management plans targeting allergies and anaphylaxis should be introduced in schools to create a safer environment for children with life-threatening allergies. Further studies on the process of implementation and the impact of such plans are also needed.

## Abbreviations

CI, Confidence Interval; SD, Standard Deviation.

## Competing interests

The authors declare that they have no competing interests.

## Authors’ contribution

NUNL participated in the study design, recruitment process, assessment of participants, data revision and analysis, data interpretation, and drafted the manuscript. LC participated in the study design, development of data collection instruments, data interpretation. LS participated in the recruitment process, assessment of participants, data interpretation. LJ participated in the study design, data analysis, data interpretation. SW participated in the study design, data interpretation. YSP performed the statistical analysis, and participated in the data interpretation. AC participated in the study design, data analysis, data interpretation. All authors read and approved the final manuscript.

## Source of Funding: The Foundations of the McGill University Health Center and the

Montreal Children’s Hospital.

The Allergy, Genes, and Environment (AllerGen) Network of Centres of Excellence.

Dr. Nguyen-Luu was supported by the Canadian Institutes of Health Research National Training Program in Allergy and Asthma and the Montreal Children’s Hospital Research Institute. Dr. Joseph and Dr. Clarke are National Scholars of the Fonds de la recherche en santé du Québec.
